# What Makes You Go Faster?: The Effect of Reward on Speeded Action under Risk

**DOI:** 10.3389/fpsyg.2017.01057

**Published:** 2017-06-26

**Authors:** Xing-jie Chen, Youngbin Kwak

**Affiliations:** Department of Psychological and Brain Sciences, University of Massachusetts Amherst, AmherstMA, United States

**Keywords:** decision making, physical effort, reward, motivation, risk-taking

## Abstract

Evaluating the potential reward and risk associated with a choice of action plays an important role in everyday decision making. However, the details behind how reward and risk affect the decisions for actions remain unclear. The present study investigates the influence of reward and risk on a decision to make a speeded motor response. One hundred and ten college students performed a Speed-Rewarded Go-NoGo task during which they were rewarded proportionally based on the speed and accuracy of their response. On each trial, the magnitude of potential reward and the probability of a forthcoming Go signal (Go-probability) were presented prior to the Go or NoGo signal. Personality traits, such as risk taking and impulsive tendencies, were measured to determine their contribution in explaining individual differences in task performance. The results showed that larger amount of rewards can motivate people to respond faster, and this effect was modulated by the assessed risk, suggesting that decisions for actions are based on a systematic trade-off between rewards and risks. Moreover, when the assessed risk was high, individuals with greater risk taking and impulsive tendencies did not adequately adjust their behavior across different reward levels. These findings shed light on the mechanistic understanding of the effect of reward and risk on decisions for a speeded action.

## Introduction

Choosing a course of action in our daily lives requires accurate assessment of the associated costs as well as the potential benefits. A typical example is shown in animal foraging behavior while they explore their environment to minimize the foraging costs and maximize retrieval of foods (MacArthur and Pianka, 1966; Kacelnik, 1997; Bautista et al., 2001). One of the most well identified costs associated with a choice of action is the effort required for the action (Wardle et al., 2011; Treadway et al., 2012; Burke et al., 2013; Hartmann et al., 2013; Klein-Flugge et al., 2015, Klein-Flügge et al., 2016). In case of the foraging example above, this is equivalent to the effort that the animal puts forth moving around from location to location to retrieve food reward.

Physical effort has often been studied in relation to intrinsic motivation and external incentive rewards (Ramnani and Miall, 2003; Ballanger et al., 2006; Mir et al., 2011; Joshua and Lisberger, 2012; Chen and Chen, 2013). These studies demonstrate that presenting potential reward outcomes can lead to faster responses and exertion of greater forces during an action required for retrieving the reward. More recent work has shown that there is a systematic trade-off between physical effort and the associated rewards in humans. Specifically these studies show that people decide to put greater physical efforts only when it will result in larger rewards (Wardle et al., 2011; Treadway et al., 2012; Burke et al., 2013; Hartmann et al., 2013; Klein-Flugge et al., 2015, Klein-Flügge et al., 2016). This suggests that similar to the temporal delay to reward arrival, physical efforts can discount the reward value at stake.

It is important to note that risk, as well as reward, is one of the key variables of decision making under uncertainty. In general terms, risk is known as a chance of negative outcome (Mishra, 2014), such as harm, loss, and danger (Leigh, 1999; Bornovalova et al., 2009). Risk is also an important variable to consider in decisions for course of actions. For example, while one may choose to drive fast to avoid being late for work, one should also consider that speed driving increases the risk of traffic accidents. Despite its relevance to real life, not many studies have focused on how risk plays a role in decisions for actions. In one study, a statistical decision theory was developed to explain the processes underlying a motor action under risk, using a simple target-hitting task (Trommershäuser et al., 2003a,b, 2005). In this task, participants were asked to rapidly hit a target area using their fingertips in order to gain a reward and received a penalty if they hit the non-target areas. Thus the risk related with their action is proportional to their motor variability. The experimental data and the model suggested that decisions on an action was made based on one’s estimate of the sensorimotor variability, which allowed controlling for their motor responses to minimize the risk associated with the movement and maximize the reward (Trommershäuser et al., 2003a). This study, however, was not designed to look at the motivational aspect of the risk-taking movements. First of all, the levels of obtainable rewards did not vary, while the magnitude of expected rewards could motivate people toward a high risk action (Doya, 2008). Furthermore, the level of risk associated with an action was not explicitly described such that one can make prior judgment on the course of action. Instead it was implicitly defined as a result of motor variability. Further studies considering both reward and risk in the same context is required to clarify the processes underlying decision making for an action.

The current study aimed to determine the effect of reward and risk on decisions for a speeded action. While “speed” is an important variable determining the characteristics of a movement, most studies have only focused on physical force in the studies of decision making for actions (e.g., Kurniawan et al., 2010; Meyniel and Pessiglione, 2014; Skvortsova et al., 2014). Movement speed is one of the most important factors influencing sensorimotor variability that is associated with risks during a movement (Trommershäuser et al., 2003b, 2005). More importantly, speed is naturally associated with greater risk for failure in any task performance as demonstrated in speed–accuracy trade-off (Pachella, 1973; Ratcliff and Tuerlinckx, 2002; Franks et al., 2003). Thus movement speed is an ideal measure to look at the effects of risk in decision making for action.

In an effort to focus on speed as a decision variable for actions, as well as to clarify the role of reward and risk, we developed a Speed-Rewarded version of the widely used Go-NoGo task. In this task, participants gain or lose points based on performance speed and accuracy. We focused on how they trade-off between speed and accuracy based on different levels of potential reward and perceived risk level associated with an action. Additionally, we investigated how personality traits such as impulsivity and risk-taking tendencies contribute to individual differences in the Speed-Rewarded Go-NoGo task. Previous studies suggest that impulsive individuals were less sensitive to negative consequences and are more willing to take risks during risky decision making tasks (Bechara et al., 1999, 2002; Bornovalova et al., 2009; Lauriola et al., 2014). We hypothesized that there would be a systematic trade-off between speed and accuracy based on the expected value of an action, which would be calculated by potential reward and perceived risk level associated with the action. Furthermore, these effects will be modulated by individual differences in impulsive and risk-taking tendencies.

## Materials and Methods

### Participants and Design

A total of 110 college students (20 males, 22.21 ± 2.13 years) without a history of psychiatric and neurological illness, or alcohol/drug dependence were recruited from University of Massachusetts, Amherst, MA, United States. All study participants signed a written informed consent in accordance with the Declaration of Helsinki, approved by the UMass Institutional Review Board before the experiment and received course credits for participation after completion of the experiment.

### Speed-Rewarded Go-NoGo Task

During the first phase of the task, participants completed a typical Go-NoGo task in which Go signals appeared 80% of the time in a total of 100 trials. Response times (RT) to the Go signals were used to calculate the RT categories for determining actual rewards in the Speed-Rewarded Go-NoGo task in the second phase. Five RT categories were determined based on the lognormal distribution of the Go signal RTs from the first phase (Category 1: RT < μ – 2σ; Category 2: μ – 2σ < RT < μ – σ; Category 3: μ – σ < RT < μ; Category 4: μ < RT < μ + σ; Category 5: RT ≥ μ + σ; μ and σ refers to the mean and standard deviation of the lognormal distribution).

**FIGURE 1:**
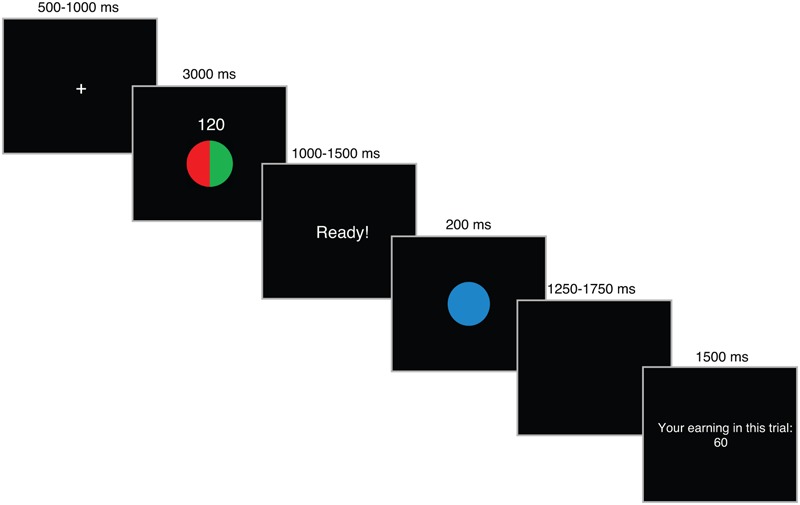
The trial structure for Speed-Rewarded Go-NoGo task.

In the second phase of the task, participants performed the Speed-Rewarded Go-NoGo task (**Figure 1**). Participants were rewarded based on the speed and accuracy of response. Throughout the task, participants were instructed to use their right index finger to press a button on a response box. A faster response to a Go signal resulted in higher rewards, whereas an incorrect response to a NoGo signal (i.e., false alarm) was punished by loss of reward points. On each trial of the task, participants were first presented with a trial information cue. The cue contained information about the amount of reward points they could earn – either 120 (high reward) or 6 (low reward) – and the probability that a Go signal would appear in that trial as described in a pie-chart (Go-probability: 20, 50, or 80%). Following the presentation of a trial information cue, the screen displayed a “Ready!” sign for a variable time window (1000–1500 ms), which prompted the participants to prepare for a response. A Go (geometric shape in blue) or NoGo (same geometric shape in gray) signal, determined by the Go signal probability, was presented in the following screen. After participant’s response, the actual reward amount that the participant won based on his/her performance was displayed. A correct response to a Go signal was rewarded based on RT using the pre-defined RT category from the first phase. For trials that meet the RT category 1, the total point at stake (either 120 or 6) is awarded. For trials that fall under RT category 2, 3, 4, and 5, points are discounted to 50, 25, 12.5, and 0% of the total point, respectively. Correct responses to a NoGo signal does not result in any rewards. However, an incorrect response to a NoGo signal (i.e., false alarm) will result in a loss of the total points at stake (i.e., results in -120 or -6). Thus, the decision to Go entails a risk for resulting in negative points. The Go-probability can therefore be considered as a metric based on which the participants can assess the risk of negative outcomes associated with the Go decision. A fixation cue was displayed during inter-trial interval. There were six blocks with 192 trials in total (32 trials in each block: four trials with low reward and 20% Go-probability; eight trials with low reward and 50% Go-probability; four trials with low reward and 80% Go-probability; four trials with high reward and 20% Go-probability; eight trials with high reward and 50% Go-probability; four trials with high reward with 80% Go-probability). After each block, participants were shown the accumulated amount of points they’ve earned up until the previous block.

### Behavioral Psychometric Measures

In an effort to determine how individual differences in personality traits related with impulsivity and risk taking contributes in performance during Speed-Rewarded Go-NoGo task, we additionally collected the following measures.

#### Behavioral Inhibition and Activation Scale (BIS/BAS)

The BIS/BAS contains 24 items and yields four factors measuring the behavioral inhibition system (BIS) and behavioral active system (Carver and White, 1994). The four factors include Drive, Fun Seeking, Reward Responsiveness, and Behavioral Inhibition. Participants are asked to rate each item with a 4-point Likert scale.

#### Barratt Impulsiveness Scale

Barratt Impulsiveness Scale is a 30 item self-report instrument designed to assess the personality/behavioral construct of impulsiveness. It has the following three factors: Factor 1 (motor impulsivity); Factor 2 (non-planning impulsiveness); Factor 3 (attentional impulsiveness) (Barratt et al., 1994). Participants are asked to rate each item with a 4-point Likert scale.

#### Gambling Related Cognitions Scale (GRCS)

Gambling Related Cognitions Scale contains 23 items in community-based population with five factors: Gambling expectancies, Illusion of control, Predictive control, Inability to stop gambling, and Interpretive bias (Raylu and Oei, 2004). Participants are asked to rate each item with a 7-point Likert scale.

#### Delay Discounting Task

The participants will choose between getting a relatively small amount of money today or getting a relatively large amount of money in the future (Kirby et al., 1999). Here is a sample question “Would you prefer $ 54 today, or $ 55 in 117 days?” There were 27 items in this task. The delay discounting rate (value *k*) in our study was fitted to Mazur’s (1987) hyperbolic equation (Myerson et al., 2014): *V = A/(1 + kD)*. This equation describes how the subjective value (*V*) of a reward (*A*) is discounted as a function of delay (*D*) (Mazur, 1987). High *k*-value indicated high delay discounting rate.

## Results

We looked at the reaction time to the Go signals and the false alarm rates (the proportion of incorrect responses to NoGo signals) in each experimental condition as displayed in **Table 1**. Since different categories for reward size were based on the standard deviation of reaction time of each participant, *Z*-scored RTs were for all the analyses. Raw RTs within each individual were log-transformed, after which they were converted into *Z*-scores.

**Table 1:** Performance of Speed-Rewarded Go-NoGo task in each condition.

		20%		50%		80%	
		*M*	*SD*	*M*	*SD*	*M*	*SD*
Low reward	FA for NoGo	0.056	0.097	0.112	0.124	0.294	0.304
	*Z*-scored RT for Go	0.465	0.520	0.290	0.424	0.026	0.512
	Speed–accuracy trade-off	0.783	0.504	0.951	0.500	1.806	1.86
High reward	FA for NoGo	0.052	0.071	0.149	0.156	0.316	0.289
	*Z*-scored RT for Go	0.348	0.454	0.073	0.234	-0.314	0.321
	Speed–accuracy trade-off	0.862	0.650	1.200	0.572	2.399	1.875

*FA, false alarm rate; RT, reaction time.*

### The Effect of Reward and Go Signal Probability

**FIGURE 2:**
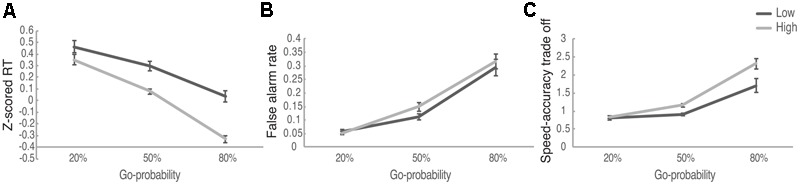
The performance of Speed-Rewarded Go-NoGo task. **(A)** *Z*-scored RT in different Reward and Go-probability conditions; **(B)** false alarm rate in different Reward and Go-probability conditions; **(C)** the speed-accuracy trade-off measure in different Reward and Go-probability conditions.

A set of 2 (Reward: High, Low) × 3 (Go-probability: 20, 50, 80%) within subject ANOVA was performed for the RT to Go signals, the false alarm rates to NoGo signals as well as the speed–accuracy trade-off measure. For RT we found a main effect of Reward [*F*(1,98) = 27.684, *p* < 0.001, η^2^ = 0.219, *M*_low_ = 0.264, *M*_high_ = 0.033] and Probability [*F*(2,196) = 88.487, *p* < 0.001, η^2^ = 0.472], as well as the interaction between Reward and Probability [*F*(2,196) = 6.572, *p* = 0.002, η^2^ = 0.062] (**Figure 2A**). *Post hoc* analysis suggested that when the Go-probability was relatively low (20%), there was no significant difference between RT for high reward compared to the low reward conditions (*p* = 0.111). When the Go-probability was 50 and 80%, RT was significantly smaller for high reward condition compared to low reward condition (both *p*s < 0.001, with Bonforroni correction). These results suggested that the effect of reward on speed was modulated by the assessed level of risk as described in the Go-probability. Speeding up for larger reward only happened when the Go-probability was 50% or above (i.e., when the risk for losing associated with false alarm was low).

For the false alarm rate, we found a significant main effect of Go-probability [*F*(2,218) = 91.872, *p* < 0.001, η^2^ = 0.457]. False alarm rate was higher in 80% probability condition (*M* = 0.305) than in 50% probability condition (*M* = 0.131), and it was higher in 50% probability condition than in 20% probability condition (*M* = 0.054) (all *p*s < 0.001, with Bonforroni correction). The main effect of reward [*F*(1,109) = 2.571, *p* > 0.10, η^2^ = 0.023, *M*_low_ = 0.154, *M*_high_ = 0.172] and the interaction between reward and probability [*F*(2,218) = 1.144, *p* > 0.10, η^2^ = 0.010] were not significant (**Figure 2B**). These results suggest that there was a greater tendency to take risks associated with a speeded Go response when there was an explicitly known low probability for losing due to false alarm (i.e., high Go signal probability).

We also determined whether reward and risk systematically influenced the speed–accuracy trade-off. The following formula as an index of speed–accuracy trade-off (Fitts, 1954): 1/RT^∗^ACC. In order to keep all the RT values positive, to be used in the speed–accuracy trade-off measure, we applied exponential function to the RT *Z*-scores. The higher value of the trade-off measure indicates that participants prefer to trade accuracy for faster response and the lower value means that participants prefer to trade speed for higher accuracy. The average speed–accuracy trade-off measure in different reward and Go-probability conditions was displayed in **Table 1**. For the speed accuracy trade-off, we found a significant main effect of reward [*F*(1,88) = 11.261, *p* = 0.001, η^2^ = 0.113] and Go-probability [*F*(2,176) = 62.506, *p* < 0.001, η^2^ = 0.415] as well as the interaction between them [*F*(2,176) = 5.09, *p* = 0.007, η^2^ = 0.055] (**Figure 2C**). The simple effect analysis suggested that when the Go-probability was 20%, there was no significant difference between high and low reward conditions (*p* = 0.77). When the Go-probability is 50% or 80%, the speed–accuracy trade-off was higher in high reward condition compared to low reward condition (for 50% Go-probability, *p* < 0.001, for 80% Go-probability, *p* = 0.007, with Bonforroni correction). Consistent with the results from RT, these results suggested that the effect of reward on movement speed was modulated by the assessed level of risk as described in the Go-probability. When the Go-probability was high (50 or 80%), the risk for losing associated with false alarm was low, participants preferred to trade off accuracy in order to response faster in order to get the high reward.

One limitation in our sample was that the ratio between female and male was disproportionate. In order to explore if there were any effect driven by gender difference, we performed additional analysis including gender as a between subject factor and performed 2 (Gender: male, female) × 2 (Reward: high, low) × 3 (Go-probability: 20, 50, 80%) mixed effect ANOVA.

For both the RT and FA as well as the speed–accuracy trade-off measure, gender didn’t show any main effects (all *p*s > 0.05) and there was no significant interaction between reward and gender (all *p*s > 0.05). Also gender didn’t interact with Go-probability for speed–accuracy trade-off [*F*(2,174) = 2.64, *p* > 0.05] and there was no significant three-way interaction between gender, reward and Go-probability for speed–accuracy trade-off. However, there was a significant interaction between gender and Go-probability for RT [*F*(2,196) = 4.024, *p* = 0.019, η^2^ = 0.039] and for the false alarm rate [*F*(2,196) = 4.917, *p* = 0.008, η^2^ = 0.044]. Additionally, there was a three-way interaction among gender, reward and Go-probability for false alarm rate [*F*(2,216) = 4.577, *p* = 0.011, η^2^ = 0.041] and RT [*F*(2,196) = 3.771, *p* = 0.025, η^2^ = 0.037]. *Post hoc* test suggested that for males, there was a significant interaction between reward and Go-probability for false alarm rate [*F*(2,216) = 3.61, *p* = 0.029] while for females, the interaction between reward and Go-probability was not significant [*F*(2,216) = 1.52, *p* > 0.1]. But for the RT, there was a significant interaction between reward and Go-probability for females [*F*(2,196) = 10.04, *p* < 0.001] while the interaction between reward and Go-probability was not significant for males [*F*(2,196) < 1, *p* > 0.1]. These results suggest that gender may play a role in the way reward and risk level influence decision making for a speeded action. Further studies are required to clarify the effect of gender with more balanced sample size between males and females.

### Contribution of Risk-Taking and Impulsive Traits in Speed-Rewarded Go-NoGo Performance

Correlation analyses were conducted between the measures of risk-taking and impulsive traits, and the performance measures of Speed-Rewarded Go-NoGo task. The results are displayed in **Tables 2, 3**. Significantly positive correlations with the false alarm rate were found in the GRCS and delay discounting (**Table 2**). Significant negative correlation with the RT was found in BIS (**Table 3**). No significant relationships were found between speed–accuracy trade-off and any of the risk-taking and impulsive trait measures.

**Table 2:** The correlations among false alarm rate, delay discounting rate and impulsive and risk-taking tendencies in each probability and reward condition.

	BIS/BAS		Barratt Impulsiveness Scale	GRCS	Delay discounting rate (*k*)
	BIS	BAS			
FA_20	-0.025	0.102	-0.089	0.177	0.218^∗^
FA_50	-0.005	-0.094	-0.118	0.15	0.117
FA_80	-0.012	0.013	-0.025	0.192^∗^	0.229^∗^
FA_Low	0.022	0.011	-0.097	0.255^∗∗^	0.256^∗∗^
FA_High	-0.045	-0.021	-0.04	0.143	0.177
FA_Total	-0.015	-0.007	-0.076	0.219^∗^	0.241^∗^

*^∗^*p* < 0.05.*

*^∗∗^*p* < 0.01.*

*FA_20; FA_50; FA_80: False alarm rate in 20, 50, 80% Go-probability conditions, FA_Low; FA_High: False alarm rate in high and low reward conditions, FA_Total: overall false alarm rate.*

**Table 3:** The correlations among normalized RT, discounting rate, and risk preference in each probability and reward condition.

	BIS/BAS		Barratt Impulsiveness Scale	GRCS	Delay discounting rate (*k*)
	BIS	BAS			
*Z*-scored RT_20	-0.067	0.09	-0.001	-0.085	0.125
*Z*-scored RT_50	-0.119	0.076	-0.11	0.061	0.125
*Z*-scored RT_80	-0.099	0.126	-0.029	-0.093	-0.077
*Z*-scored RT_Low	-0.184	0.203^∗^	-0.139	-0.08	0.04
*Z*-scored RT_High	0.035	-0.111	0.105	0.078	0.125
*Z*-scored RT_Total	-0.199^∗^	0.177	-0.101	-0.129	0.103

*^∗^*p* < 0.05.*

*^∗∗^*p* < 0.01.*

**FIGURE 3:**
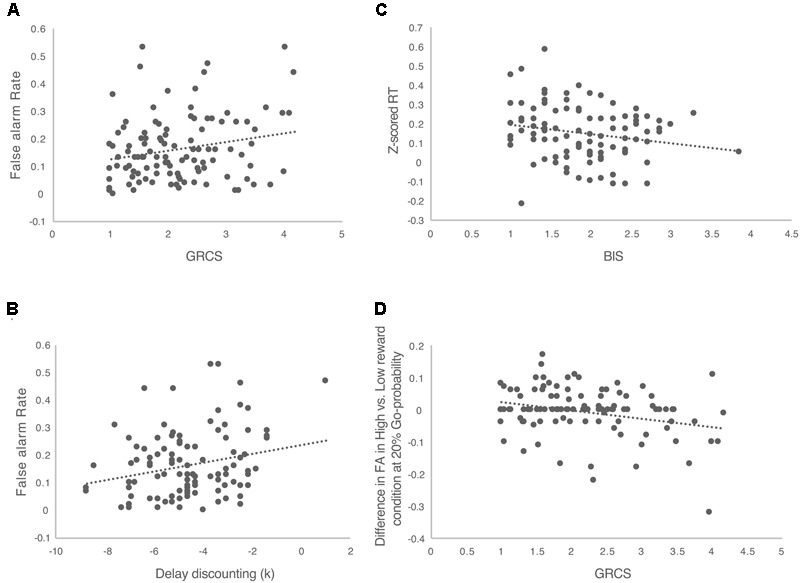
The correlations between the performance of Speed-Rewarded Go-NoGo task and the impulsive and risk-taking tendencies. **(A)** The correlation between GRCS total score and false alarm rate; **(B)** the correlation between delay discounting and false alarm rate; **(C)** the correlation between BIS score and *Z*-scored RT; **(D)** the correlation between the GRCS total score and the difference of false alarm rate between high and low reward conditions.

In general, the overall false alarm rate was positively correlated with the total score of GRCS (*r* = 0.219, *p* = 0.023, **Figure 3A**). This suggested that people with higher gambling-oriented cognition style have greater tendency to take risks. Further correlation analyses were conducted between GRCS and the false alarm rates in different reward and probability conditions. The results suggested that in the high probability condition (80%), there was a significant correlation between false alarm rate and the total score of GRCS (*r* = 0.192, *p* = 0.046). But in the 20 and 50% probability conditions, there were no significant correlations. Also with low reward, there was significant correlation between the false alarm rate and the total score of GRCS (*r* = 0.255, *p* = 0.008). But no significant correlation was found with high reward condition.

The delay-discounting rate was significantly correlated with the overall false alarm rate (*r* = 0.241, *p* = 0.013, **Figure 3B**), indicating that individuals with larger delay discounting rate, took more risks. Across different Go signal probability conditions significant correlations were found in 20% (*r* = 0.218, *p* = 0.024) and 80% (*r* = 0.229, *p* = 0.018) probability conditions. No significant correlation was found in the 50% probability condition. Across different reward levels, in the low reward condition, there was a significant correlation between false alarm rate and delay discounting rate (*r* = 0.256, *p* = 0.008). No significant correlation was found with high reward condition.

There was a significant negative correlation between the RT and BIS in BIS/BAS (*r* = -0.199, *p* = 0.05, **Figure 3C**), indicating that individuals with greater behavioral avoidance (behavioral inhibition system) would response faster. Further correlation analyses were conducted between BIS/BAS and the RT in different reward and probability conditions. For BIS subscale, we didn’t find any significant correlations across different reward and Go-probability conditions. But there was a positive correlation between BAS and the RT in low reward condition (*r* = 0.203, *p* = 0.047). This suggested that individuals with greater behavioral approach system (behavioral activation system) would response slower in low reward condition. But no significant correlation was found in high reward condition as well as the different Go-probability conditions.

In an effort to determine whether the relationship with the risk-taking and impulsive trait measures differently change across reward level Go-probabilities, we calculated the difference in false alarm rate between high and low reward conditions separately in each probability condition and looked at the correlation between this difference measure with the risk-taking and impulsive trait measures. The results showed that in the 20% probability condition, the difference of false alarm rate between the high and low reward conditions was negatively correlated with total score of GRCS (*r* = -0.281, *p* = 0.003, **Figure 3D**). With 50 and 80% probability conditions, no significant correlations were found. This indicated that the effect of reward on increasing false alarm rate was greater for people who demonstrated less gambling oriented cognition styles and that this effect was specific when the Go signal probability was low. No significant correlations were found with the delay-discounting rate and BIS/BAS.

## Discussion

The current study aimed to explore the effect of reward and risk on decisions for speeded actions. To this end, we developed a task paradigm in which we rewarded speeded motor responses, while at the same time manipulating the risks associated with the action. Specifically, in our task, faster responses would result in higher rewards while at the same time it also entails a higher risk of losing rewards due to false alarm. Our results showed that higher rewards motivated people to respond faster and this effect was modulated by the Go signal probability, which explicitly influenced the perceived risk associated with the action. Specifically, when the probability of Go signals was relatively high (50 and 80%), the higher rewards led to significantly faster response to Go signals whereas the modulatory effect of reward was not significant when the probability of Go signals was low (20%). More importantly as shown by our results of the speed–accuracy trade-off measure, there was a greater sacrifice for accuracy in favor of speed when the response was associated with higher potential reward and when the perceived risk level was low (i.e., higher Go-probability). These results suggest that decisions for a speeded action is determined by a systematic trade-off between cost and benefit associated with an action, which is based on the potential reward and risk level, the two determinants of the action value.

These results are in line with previous studies showing the powerful motivational role of monetary rewards in the conscious selection of actions (Ballanger et al., 2006; Kurniawan et al., 2010; Meyniel and Pessiglione, 2014; Skvortsova et al., 2014). In the present study, the level of potential rewards was presented as either high or low and the actual amount of reward was proportionally deducted from the potential reward based on the speed of the response. As expected, higher rewards resulted in faster responses suggesting an increase in motivation. Faster responses, however, were inevitably associated with higher risk of incorrect responses as generally depicted in speed–accuracy trade-off (Fitts, 1954), which is readily acknowledged in our everyday decision making as implied in the idiom “Haste makes waste.” In our task, we formalized the risk associated with speed by imposing a loss of points when there’s a false alarm, a feature that adds on an ecological validity to our task.

The unique aspect of the current study is that we tried to understand how the trade-off between rewards and costs are manifested as a trade-off between speed and accuracy across different reward and risk levels. While there have been studies that investigate the mechanisms underlying the trade-offs between physical effort and reward (Skvortsova et al., 2014; Scholl et al., 2015; Harris and Lim, 2016; Klein-Flügge et al., 2016; Le Bouc et al., 2016), not many studies have looked at whether similar trade-off appears for movement speed. Furthermore, these previous studies have not considered the effect of risk associated with an action. Risk, as well as reward amount and delay to reward delivery, is a key variable of economic decision making under uncertainty (Doya, 2008; Platt and Huettel, 2008). Our study showed that decisions on speeded actions are also made based on the potential rewards to gain as well as the associated risk level similar to the way economic choices are made. Specifically, as preparing for a faster response introduces higher risk of failure to inhibit the action which may result in loss of points, participants only decided to speed up when the known probability of losing is low (i.e., higher Go signal probability).

In order to determine whether the degree to which reward and risk level are taken into consideration systematically varies across individuals, we investigated how independent measures of risk taking and impulsive traits explain performance in our task. Our results showed that in general, greater false alarm rate was associated with the greater tendency of risk taking and impulsive traits as reflected in higher delay discounting rate and higher gambling oriented cognition style. Interestingly, when we separated out the trials into either different levels of rewards or different levels of Go signal probability, these effects were observed only when the potential reward was low or when the probability of Go signal was either high (80%) or low (20%). This may suggest that the relationship between task performance and the risk-taking and impulsive tendencies are present specifically when there is less conflict associated with a decision for an action; when it is easier to make the decision to Go or NoGo. We also looked at the degree to which the reward level affected performance at each Go signal probability and found that the difference in false alarm rate between high vs. low reward conditions was negatively correlated with the gambling oriented cognition style, only when the Go signal probability was low (i.e., 20%). This suggests that particularly in the case of high assessed-risk, individuals who are more prone to gambling are less capable of adjusting their behaviors based on the level of reward level compared to individuals who are less prone to gambling. We also found association between RT the general motivation system measured by BIS/BAS, suggesting faster responses in individual with great behavioral inhibition system (BIS). Together, these results suggest that individual differences in motivational system as well as risk-taking and impulsive tendencies modulate how one computes the value of an action based on potential reward and assessed risk level associated with the speeded action.

The results of our study can also be explained in terms of the underlying neural circuitry. Specifically, the direct and indirect pathways from the striatum to the basal ganglia output nuclei (the internal globus pallidus and the substantia nigra pars reticulate) are known to be the canonical model neural circuitry involved in the initiation or inhibition of motor actions (Calabresi et al., 2014). Whereas the direct pathway promotes movement, the indirect pathway inhibits an action. More recently the hyperdirect pathway, which directly connects the cortex to the subthalamic nucleus has been identified to serve a critical role in suppressing erroneous actions (Calabresi et al., 2014; Jahanshahi et al., 2015). It is the balanced interaction between these pathways that leads to appropriate initiation and inhibition of motor plans (Aron, 2011; Dunovan et al., 2015). In our study, the motor plan for initiation or inhibition of the motor response is influenced by the reward and Go-probability. Thus it is likely that this information will shape the interaction within the basal ganglia circuitry to modulate the motor plan for the Go vs. NoGo for the upcoming trial.

In summary, our study investigated the contribution of the reward amount and assessed risk level in decision making for speeded actions. We used a novel experimental paradigm by presenting an ecologically valid decision making scenario, which implements both reward and risk during a Go-NoGo task. Our results indicate that in general, larger rewards increased movement speed despite being associated with higher risk of losing and the degree to which reward influenced performance, was modulated by the assessed risk-level. This was reflected as a systematic speed–accuracy trade-off across different levels of reward and risk, which are the two determinants of the action value. Moreover, individual differences in risk taking and impulsive tendencies contributed to this process such that individuals with greater risk taking and impulsive tendencies did not adequately adjust their behavior across different reward levels, particularly in the case of high assessed risk-level. Our results demonstrate that when making decisions for a speeded action, the associated costs and benefits are evaluated based on the potential reward and risk level, which are the two determinants of the action value. This suggests that the decisions for speeded actions are made in a similar manner to decisions about economic choices integrating equivalent decision variables into trade-offs for optimal outcome. Future research investigating the neural underpinnings of the behavioral effects will lead to a mechanistic understanding of how reward and risk influence decisions for a speeded action, which intrinsically possess risk such as what’s framed as the speed–accuracy trade-off. Moreover, considering the gender difference in false alarm rate and speed–accuracy trade-off in the present study, future studies are required to clarify the effect of gender with more balanced sample size between males and females.

## Author Contributions

X-jC and YK designed the study, collected and analyzed data and prepared the manuscript.

## Conflict of Interest Statement

The authors declare that the research was conducted in the absence of any commercial or financial relationships that could be construed as a potential conflict of interest.
